# The GADD45G/p38 MAPK/CDC25B signaling pathway enhances neurite outgrowth by promoting microtubule polymerization

**DOI:** 10.1016/j.isci.2022.104089

**Published:** 2022-04-04

**Authors:** Yoshitaka Kase, Tsukika Sato, Yuji Okano, Hideyuki Okano

**Affiliations:** 1 Department of Physiology, Keio University School of Medicine, 35 Shinanomachi, Shinjuku-ku, Tokyo 160-8582, Japan; 2 Department of Geriatric Medicine, Graduate School of Medicine, The University of Tokyo, 7-3-1 Hongo, Bunkyo-ku, Tokyo 113-8655, Japan

**Keywords:** Biological sciences, Neuroscience, Cellular neuroscience, Cell biology

## Abstract

GADD45G, one of the genes containing the human-specific conserved deletion enhancer-sequence (hCONDEL), has contributed to the evolution of the human cerebrum, but its function in human neurons has not been established. Here, we show that the GADD45G/p38 MAPK/CDC25B signaling pathway promotes neurite outgrowth in human neurons by facilitating microtubule polymerization. This pathway ultimately promotes dephosphorylation of phosphorylated CRMP2 which in turn promotes microtubule assembly. We also found that *GADD45G* was highly expressed in developing human cerebral specimens. In addition, RK-682, which is the inhibitor of a phosphatase of p38 MAPK and was found in *Streptomyces* sp., was shown to promote microtubule polymerization and neurite outgrowth by enhancing p38 MAPK/CDC25B signaling. These *in vitro* and *in vivo* results indicate that GADD45G/p38 MAPK/CDC25B enhances neurite outgrowth in human neurons.

## Highlights

• The GADD45G/p38/CDC25B signaling pathway promotes neurite outgrowth in human neurons
• The GADD45G/p38/CDC25B signaling pathway ultimately promotes microtubule assembly
• RK-682 promote neurite outgrowth by enhancing p38 MAPK/CDC25B signaling

## Graphical abstract

**Figure:**
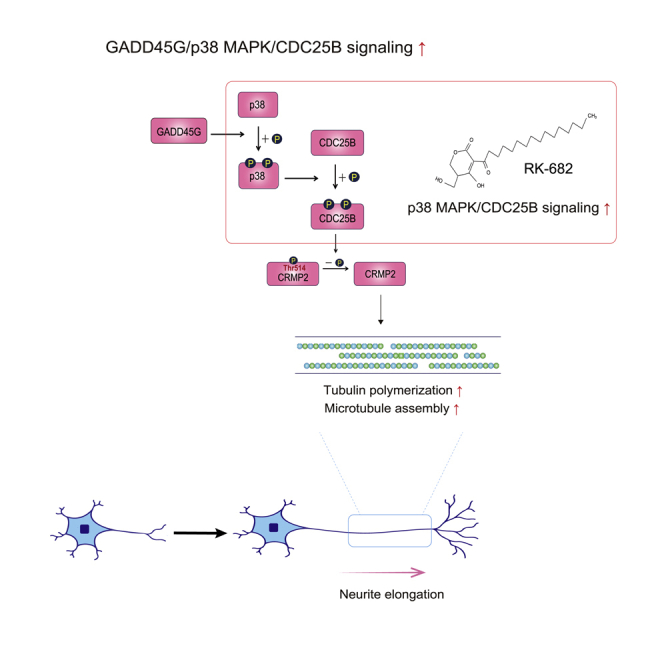

## Introduction

The human-specific conserved deletion sequences hCONDEL is a category of regulatory DNA sequences including enhancers that are deleted specifically in humans but are present in the genome of chimpanzees and other mammalian species (McLean et al., 2011). One of the hCONDEL members is the tumor suppressor gene, growth arrest, and DNA damage inducible gamma (*GADD45G*). In addition to being a tumor suppressor gene, *GADD45G* has been implicated in the evolution of the human neocortex, and it is known to be involved in sex determination through p38 MAPK signaling (Gierl et al., 2012). *GADD45G* also regulates cell proliferation via p38 MAPK-mediated regulation of p21 (Ishida et al., 2013).

In the present study, we focused on the functional role of *GADD45G,* which is strongly expressed in neural stem/progenitor cells (NS/PCs) and immature neurons in the developing human fetal brains. Although it is generally known as a tumor suppressor, its neuronal functions are not yet completely established. To investigate its roles in developing human brains, we took advantage of the neuronal differentiation systems of human induced pluripotent stem cell (hiPSC)-derived neural stem/progenitor cells (hiPSC-NS/PCs; neurospheres).

Here, we discovered a new signaling cascade, focused on *GADD45G*, which extends neurites by promoting the polymerization of microtubules in human neurons. We treated neurospheres with γ-secretase inhibitors (GSIs) to induce the expression of neuronal lineage genes.

In our recently initiated clinical study using hiPSC-derived NS/PCs for spinal cord injury, these cells were pretreated with GSIs before transplantation (Sugai et al., 2021). GSIs inhibit the cleavage of Notch by γ-secretase and suppress the formation of the Notch intracellular domain (NICD). The NICD induces *HES family* gene expression, which suppresses neuronal gene expression. Therefore, GSIs promote the differentiation of neural stem cells into neurons (Kageyama et al., 2007; Nelson et al., 2007; Ohtsuka, 1999).

In our preclinical studies (Okubo et al., 2016, 2018), GSI treatment has also been shown to induce proliferative cells to undergo neural differentiation and to enhance the maturation and neurite extension of transplanted neurons, thereby enhancing the therapeutic effect of cell transplantation. However, the underlying detailed mechanism remains to be clarified. In this study, we analyzed these neurospheres with RNA sequencing (RNA-seq) to identify signals involved in promoting neurite outgrowth.

We propose a novel mechanism by which the GADD45G/p38 MAPK/CDC25B signaling pathway reduces the phosphorylated (phospho-) CRMP2 level and promotes tubulin polymerization, assisting microtubule assembly and leading to neurite outgrowth of human neuronal cells. During neural network formation, especially in the human fetal brain, active neurite outgrowth is essential for brain development. Neurite elongation first requires tubulin polymerization to build microtubules that form the neuronal cytoskeleton.

Tubulins, a group of proteins that constitute microtubules, are the most abundant proteins in neuritis; besides, tubulin polymerization is an essential process in axon elongation (Witte et al., 2008). Collapsin response mediator protein 2 (CRMP2), a protein that regulates axon elongation, binds to tubulin and promotes microtubule polymerization (Fukata et al., 2002). In addition, positive and negative regulatory mechanisms upstream hierarchically regulate CRMP2 activity and microtubule polymerization. First, Sema3A receptors, neuropilin-1 (NP-1) and plexin A (PlexA), activate Rac1, which affects downstream kinases and ultimately activates activated glycogen synthase kinase 3 (GSK3β), resulting in phosphorylation of CRMP2 at Thr514 (Yoshimura et al., 2005), thereby inactivating it by weakening the affinity to tubulin (Hensley et al., 2011; Yoshimura et al., 2005). On the other hand, phosphatidylinositol-3,4,5-triphosphate (PIP3) activates Akt, which phosphorylates and inactivates GSK3β; this pathway promotes microtubule polymerization (Dajani et al., 2001; Ménager et al., 2004). Microtubule-associated proteins (MAPs) have also been reported to support microtubule polymerization by suppressing catastrophe, an event that occurs during the transition from polymerization to depolymerization (Itoh and Hotani, 1994; Roll-Mecak, 2020).

As regards the mechanism of neurite outgrowth, it is mainly mediated by promotion of microtubule polymerization. However, the signaling pathways that promote the microtubule polymerization remain largely unidentified. To date, investigations on the regulation of neurite outgrowth have mainly focused on zebrafish, mouse, rat, and chicken neurons. However, very few research works have been performed on the regulations of neurite outgrowth in human neurons. In the present study, by taking advantage of the hiPSC-NS/PCs differentiation system, we first demonstrate the novel cascade, GADD45G/p38MAPK/CDC25B, as a critical regulator of neurite outgrowth in human neurons.

## Results

### Generation of neurospheres (hiPSC-NS/PCs) that express neuronal lineage genes to identify factors that facilitate neurite outgrowth

First, we performed screening experiments to identify signals that promoted neurite outgrowth by using neurospheres. To ensure the scientific validity of the findings, we prepared two types of hiPSC-NS/PCs derived from different human iPSC lines, 201B7 (Takahashi et al., 2007) and 414C2 (Okita et al., 2011).

**Figure 1:**
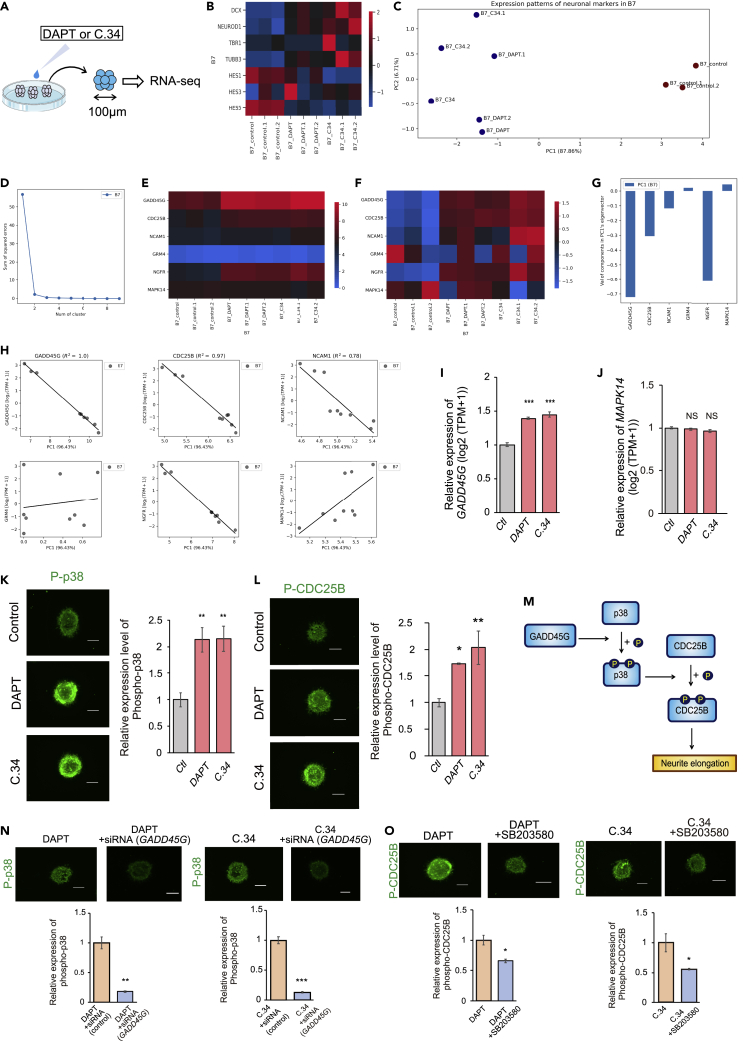
Screening for neurite-extending factors (A) Neurospheres (hiPSC-NS/PCs) approximately 100 μm in diameter were treated with DAPT or Compound 34 and analyzed by RNA-seq. (B) Heatmap of neuronal lineage-related genes showing that DAPT and Compound 34 induced neuronal gene expression in neurospheres (derived from the 201B7 line). (C and D) A scatterplot of the expression patterns of neuronal lineage-related genes that were decomposed by PCA is shown in (C). Each point was classified by k-means clustering, and the color of the point represents the cluster. Figure 1C suggests that the cells with DAPT or Compound 34 had similar expression patterns of neuronal lineage-related genes that were different from those of the control cells. In (D), the horizontal axis shows the number of clusters, and the vertical axis shows the sum of squared errors between the cluster centroids and each plot. The “elbow” point, at which the line chart bends and plateaus, suggests that the optimal number of clusters is 2. (E and F) In hiPSC-NS/PCs (derived from the 201B7 line) treated with DAPT or Compound 34 and control hiPSC-NS/PCs, MAPK-related genes were extracted from among all fluctuating genes, and heat maps of their expression levels are shown. The color of each square represents the log_2_(TPM+1) value in (E) and the Z-score value calculated from the log_2_(TPM+1) value of a single gene among all the samples in (F). (G) The log_2_(TPM+1) values of the genes listed in (E and F) in the hiPSC-NS/PCs (derived from the 201B7 line) were decomposed by PCA, and the feature value was extracted as principal component 1 (PC1). (G) shows the components in the eigenvector of PC1 and suggests that *GADD45G* is the top representative gene for PC1, which explains 96.3% of the total information in the expression matrix. (H) Linear regression models for each gene listed in (E and F). The objective variables (shown on the vertical axis) are the log_2_(TPM+1) values, the explanatory variables (shown in the horizontal axis) are the PC1 values calculated in (G), and the *R*^*2*^ values are the coefficients of determination. The fact that the linear regression model of PC1 fit the well (*R*^*2*^ > 0.26) for *GADD45G, CDC25B, NCAM1, NGFR,* and *MAPK14* suggested that the expression patterns of *GADD45G* highly resembled PC1, which explained 96.3% of the total information of the MAPK-related gene expression matrix. (I) The expression (transcript levels) of *GADD45G* in hiPSC-NS/PCs derived from hiPSCs (201B7) treated with DAPT or Compound 34 was quantified (n = 3; p < 0.001). (J) Expression (transcript levels) of *MAPK14* in hiPSC-NS/PCs (201B7) treated with DAPT or Compound 34 (201B7; n = 3; p = 0.695, p = 0.200) (414C2; n = 3; p = 0.937, p = 0.935). The expression of *MAPK14* itself was not significantly changed. (K) Immunostaining images of phospho-p38 in hiPSC-NS/PCs (201B7) treated with DAPT or Compound 34 and relative comparison of the phospho-p38 expression levels in hiPSC-NS/PCs (201B7) treated with GSIs (n = 3; p = 0.019, p = 0.018). 10 neurospheres were observed for each experiment. (L) Immunostaining images of phospho-CDC25B in hiPSC-NS/PCs (201B7) treated with DAPT or Compound 34 and relative comparison of the phospho-CDC25B expression levels in hiPSC-NS/PCs (201B7) treated with GSIs (n = 3; p = 0.049, p = 0.0018). 10 neurospheres were observed for each experiment. (M) Conceptual diagram of the pathway for neurite extension. (N) SiRNA-mediated knockdown of *GADD45G* reduced the expression of phospho-p38 even when DAPT or Compound 34 was added. Immunostaining images and a quantitative graph are shown (n = 3; p = 0.0144, p = 0.000150). 10 neurospheres were observed for each experiment. (O) Immunostaining images and a quantitative graph of phospho-CDC25B expression in cells treated with both DAPT and SB203580 (or both Compound 34 and SB203580) and in cells treated with only DAPT (or Compound 34). The expression of phospho-CDC25B was decreased in the SB203580-supplemented group (n = 3; p = 0.0168, p = 0.0436). 10 neurospheres were observed for each experiment.Statistical analyses were performed with one-way ANOVA and Tukey-Kramer post hoc tests (I, J, K, L) or unpaired two-tailed Student’s t-tests (N, O). The values in the bar graphs represent the means ± SEs. ∗p < 0.05, ∗∗p < 0.01, ∗∗∗p < 0.001. NS: not significant. Ctl: control, (C)34: Compound 34. Scale bars: 100 μm.

Treating the neurospheres with GSIs, such as DAPT (Dovey et al., 2001) or Compound 34 (Churcher et al., 2003), enhanced neuronal gene expression, and GSI-treated single neurospheres (approximately 100 μm in diameter) were used for screening (RNA-seq) (n = 3) (Figure 1A).

GSIs are known to inhibit the cleavage of Notch, a transmembrane cell surface receptor, and suppress the subsequent expression of *HES* family members (Kageyama et al., 2007; Ohtsuka, 1999), thereby enhancing neuronal lineage-related gene expression (Nelson et al., 2007). GSI treatment decreased the expression of the *HES* family and upregulated the expression of neural marker genes, such as doublecortin (*DCX*) (Gleeson et al., 1999), neurogenic differentiation 1 (*NEUROD1*) (Gao et al., 2009), T-box brain transcription factor 1 (*TBR1*) (Bedogni et al., 2010), and tubulin beta three class III (*TUBB3*) (Sullivan and Cleveland, 1986) (Figures 1B and S1A).

To evaluate the effect of GSIs on neuronal lineage-related gene induction, we examined the transcriptomes of neurospheres in the presence or absence of GSIs. We reduced the dimensionality of the gene expression matrix by principal component analysis (PCA) and performed k-means clustering to classify each data point into as many clusters as the optimal number (k = 2) (Figures 1C and S1B), which was determined by the elbow method (Figures 1D and S1C). The two kinds of GSIs, DAPT and Compound 34, showed similar effects on both types of neurospheres derived from two different human iPSC lines (201B7 and 414C2) because all the samples in the treated group (treated with DAPT or Compound 34) were classified into the same cluster that was separate from the control group (treated with DMSO) cluster (Figures 1C and S1B).

### GADD45G/p38 MAPK/CDC25B is a candidate signaling pathway that causes neurite outgrowth

Our previous report suggested that treatment of hiPSC-NS/PCs with a GSI (DAPT) altered mitogen-activated protein kinase (MAPK) signaling and induced neurite extension (Okubo et al., 2018). Therefore, among all the genes whose expression fluctuated after the administration of DAPT or Compound 34, those related to MAPK were selected and further analyzed. The expression of growth arrest and DNA damage gamma (*GADD45G*) was increased to the greatest extent (Figures 1E, 1F, S1D, and S1E), and it was also confirmed that *GADD45G* is a suitable representative for overall MAPK-related genes (Figures 1G, 1H, S1F, and S1G). The expression of *GADD45G* was significantly increased, as shown by the transcriptome data obtained from RNA-seq analysis (Figures 1I and S1H). Moreover, among all the genes whose expression was altered by treatment with DAPT or Compound 34, *GADD45G* exhibited the most elevated expression (Table S1).

In addition, we reviewed previous reports and focused on *GADD45G*, p38 MAPK, and cell division cycle 25B (CDC25B) (Cha et al., 2007; Gierl et al., 2012; Mita et al., 2002; Thalheimer et al., 2014). Although the expression of *MAPK14*, a p38 coding gene, and the total amount of p38 protein were not significantly different between the GSI-treated groups and the control groups (Figures 1J, S1I, and S2), phospho-p38 was significantly upregulated in the GSI-treated groups (Figure 1K). In addition, there were significant increases in phospho-CDC25B expression among the GSI-treated groups (Figure 1L).

Moreover, some reports have noted that in other tissues, GADD45G phosphorylates p38 MAPK (Gierl et al., 2012; Mita et al., 2002; Thalheimer et al., 2014) and phospho-p38 phosphorylates CDC25B (Cha et al., 2007). Therefore, we hypothesized that the signaling pathway cascade shown in Figure 1M is involved in neurite outgrowth.

### GADD45G phosphorylates p38 MAPK and phospho-p38 phosphorylates CDC25B

To verify whether GADD45, p38 MAPK, and CDC25B, which show changes after GSI treatment, act in the same signaling pathway (Figure 1M), several verification experiments were performed on neurospheres (hiPSC-NS/PCs).

First, siRNA-mediated knockdown of *GADD45G* significantly reduced the phospho-p38 level despite the treatment of neurospheres with GSIs (Figure 1N), indicating that GADD45G functions upstream of phospho-p38.

Next, the phospho-CDC25B level in neurospheres was suppressed by SB203580 (a p38 MAPK inhibitor) despite GSI treatments (Figure 1O). These results showed that the signaling pathway shown in Figure 1M was established.

### GADD45G/p38 MAPK/CDC25B signaling promotes microtubule polymerization by reducing the phospho-CRMP2 level

Because the experiments described above revealed that these molecules act in a single signaling pathway, we then tested whether activation of this signaling pathway leads to neurite outgrowth.

**Figure 2:**
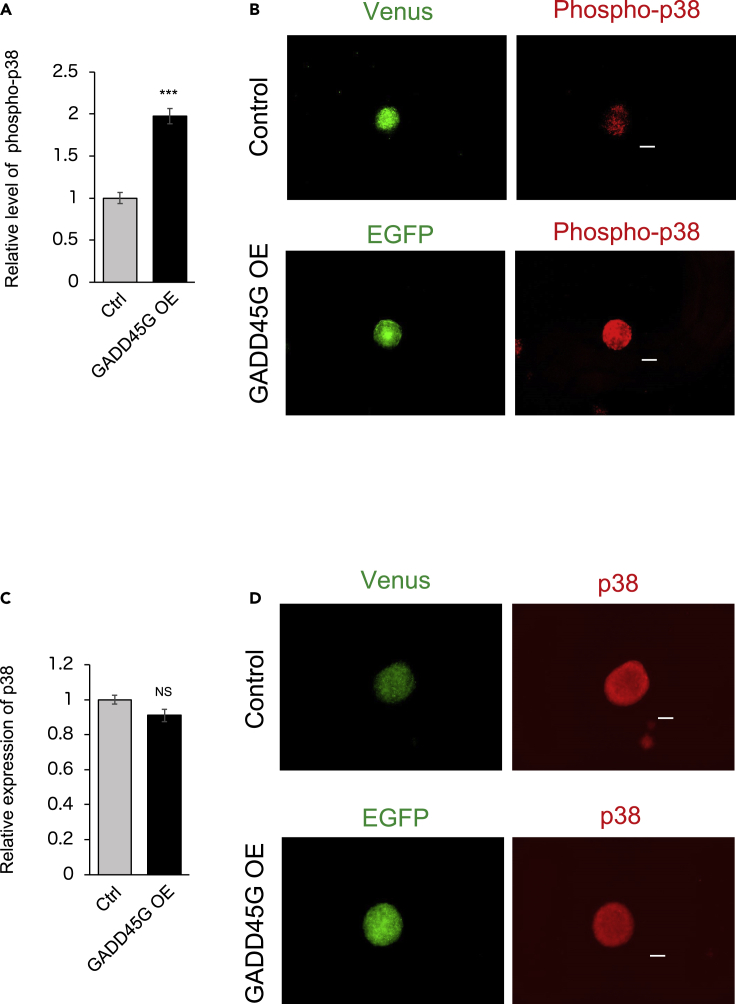
The phosphorylation level of p38 is increased in the neurosphere derived from the hiPSC (201B7) overexpressing GADD45G (A) Quantification of the expression level of phosphorylated p38 when GADD45G is overexpressed in the hiPSC (201B7)-derived neurospheres with a lentiviral vector (n = 3, p = 0.000936). (B) Immunostaining images of phosphorylated p38 in the neurosphere derived from the hiPSC (201B7) overexpressing GADD45G. Venus is used as a reporter in the control lentiviral vector and EGFP is used as a reporter in the GADD45G overexpressing lentiviral vector. (C) Quantification of the expression level of total p38 when GADD45G is overexpressed in the hiPSC (201B7)-derived neurospheres with a lentiviral vector (n = 3, p = 0.107). (D) Immunostaining images of total p38 in the neurosphere derived from the hiPSC (201B7) overexpressing GADD45G. Venus is used as a reporter in the control lentiviral vector and EGFP is used as a reporter in the GADD45G overexpressing lentiviral vector.Statistical analyses were performed with unpaired two-tailed Student’s t-tests. The values in the bar graphs represent the means ± SEs. 10 neurospheres were observed for each experiment. ∗∗∗p < 0.001. Scale bars: 100 μm.

**Figure 3:**
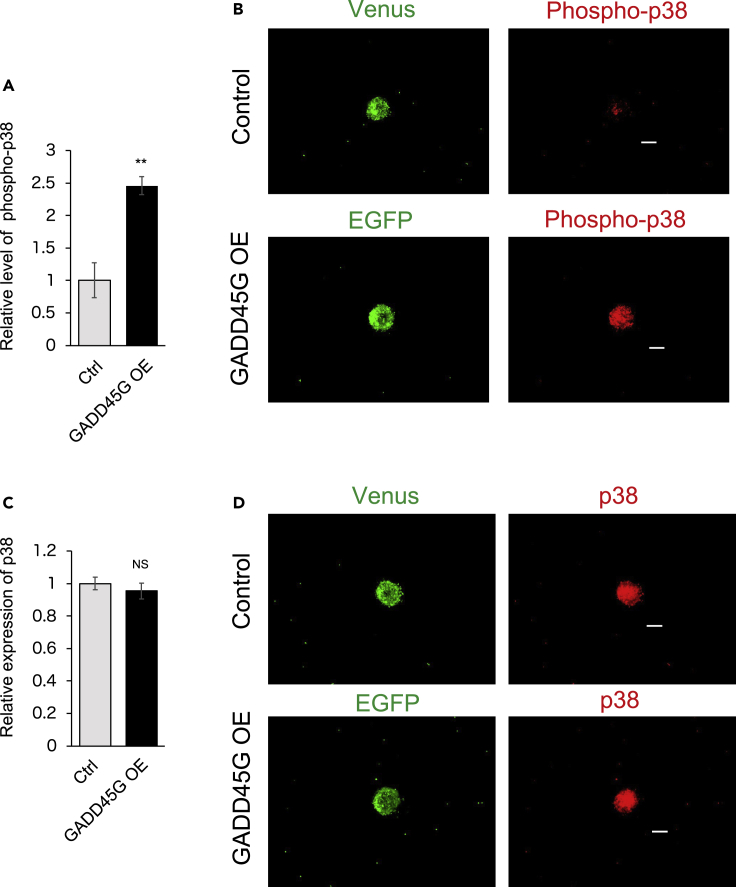
The phosphorylation level of p38 is increased in the neurosphere derived from the hiPSC (414C2) overexpressing GADD45G (A) Quantification of the expression level of phosphorylated p38 when GADD45G is overexpressed in the 414C2-derived neurospheres with a lentiviral vector (n = 3, 0.00850). (B) Immunostaining images of phosphorylated p38 in the neurosphere derived from the hiPSC (414C2) overexpressing GADD45G. Venus is used as a reporter in the control lentiviral vector and EGFP is used as a reporter in the GADD45G overexpressing lentiviral vector. (C) Quantification of the expression level of total p38 when GADD45G is overexpressed in the hiPSC (414C2)-derived neurospheres with a lentiviral vector (n = 3, 0.500). (D) Immunostaining images of total p38 in the neurosphere derived from the hiPSC (414C2) overexpressing GADD45G. Venus is used as a reporter in the control lentiviral vector and EGFP is used as a reporter in the GADD45G overexpressing lentiviral vector.Statistical analyses were performed with unpaired two-tailed Student’s t-tests. The values in the bar graphs represent the means ± SEs. 10 neurospheres were observed for each experiment. ∗∗∗p < 0.001. Scale bars: 100 μm.

First, GADD45G was overexpressed in the hiPSC (201B7)-derived neurospheres and hiPSC (414C2)-derived neurospheres using a lentiviral vector. The total p38 expression did not differ between the control and GADD45G-overexpressed neurospheres, but the phosphorylation level of p38 was significantly increased (Figures 2 and 3).

**Figure 4:**
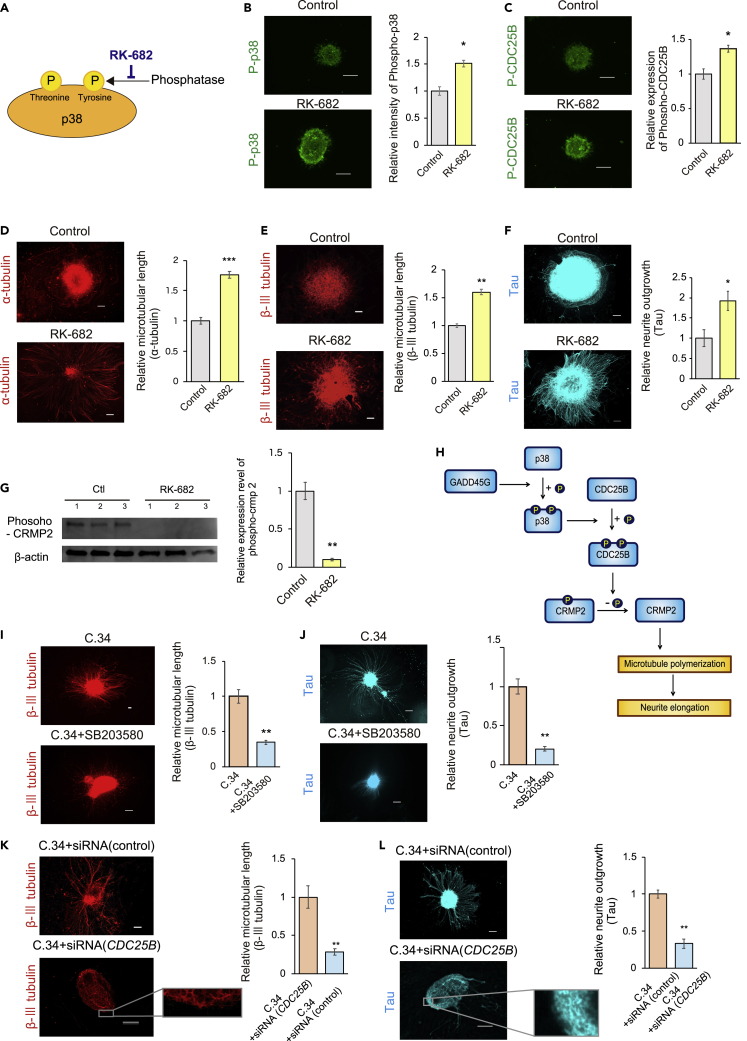
GADD45G/p38 MAPK/CDC25B signaling dephosphorylates phospho-CRMP2 and elongates neurites (A) Conceptual diagram depicting the inhibition of p38 tyrosine dephosphorylation by RK-682. (B) Immunostaining images and a quantitative graph showing that neurospheres treated with RK-682 expressed phospho-p38 at higher levels than those not treated with RK-682 (n = 3, p = 0.0319). (C) Immunostaining images and quantitative graph showing that neurospheres treated with RK-682 expressed phospho-CDC25B at higher levels than those not treated with RK-682 (n = 3; p = 0.0162). (D) Immunostaining images of α-tubulin and a quantitative graph of the microtubule length. The length was greater in the RK-682-treated group than in the untreated group (n = 3; p = 0.000635). (E) Immunostaining images of β-III tubulin and a quantitative graph of the microtubule length. The length was greater in the RK-682-treated group than in the untreated group. (F) Immunostaining images of tau and a quantitative graph of neurites. Neurites were longer in the RK-682-treated group than in the untreated group (n = 3; p = 0.0434). (G) The expression level of phospho-CRMP2 was quantified by western blotting in neurons supplemented with RK-682 and neurons not supplemented with RK-682. (H) Conceptual diagram of the signaling pathway that causes neurite outgrowth. Starting with GADD45G, dephosphorylation of CRMP2 occurs via phosphorylation of p38 MAPK and CDC25B, which promotes the polymerization of microtubules and causes neurite outgrowth. (I and J) Neurospheres treated with only Compound 34 and with both Compound 34 and SB203580 were cultured for 14 days. Differentiated neurons were immunostained with β-III tubulin (I) or tau (J). The lengths of microtubules and neurites were compared between the SB203580-treated group and the untreated group. The extension of neurites was suppressed in the SB203580 group (n = 3; p = 0.00270, p = 0.00122). (K and L) The lengths of microtubules and neurites were not extended by the siRNA-mediated knockdown of *CDC25B*. After neuronal differentiation, immunostaining of β-III tubulin (K) or tau (L) was performed. Neuronal differentiation was not inhibited even in the *CDC25B* knockdown group (high-magnification image). Neurite elongation was inhibited in the *CDC25B* knockdown group (n = 3; p = 0.00994, p = 0.00286).Statistical analyses were performed with unpaired two-tailed Student’s t-tests. The values in the bar graphs represent the means ± SEs. 10 neurospheres were observed for each experiment. ∗p < 0.05, ∗∗p < 0.01, ∗∗∗p < 0.001. (C)34: Compound 34. Scale bars: 100 μm. The neurospheres were derived from the iPSC line 201B7.

For the activation method, we also chose a compound, RK-682, because we were considering its future clinical application. RK-682 isolated from *Streptomyces* sp. can prevent tyrosine dephosphorylation of p38 (Hamaguchi et al., 1995). RK-682 prevented the dephosphorylation of phospho-p38 (Figure 4A), leading to maintenance of the activity of p38. In addition, RK-682 does not inhibit the dephosphorylation of CDC25B (Hamaguchi et al., 1995).

The phospho-p38 level was measured after RK-682 was added to hiPSC-NS/PCs and the neurospheres were cultured for approximately 24 h. The levels of phospho-p38 (Figures 4B and S3) and phospho-CDC25B (Figure 4C) were significantly increased after RK-682 treatment.

Furthermore, when neurospheres supplemented with RK-682 were cultured for 14 days to differentiate from neurospheres into neurons, the microtubule subunit, a heterodimer of α-tubulin and β-tubulin, was elongated by the addition of RK-682, which was confirmed by immunostaining of α-tubulin and β-III tubulin (Roll-Mecak, 2020) (Figures 4D and 4E). Immunostaining images of tau also revealed that the neurites were also elongated (Figure 4F). Similarly, overexpression of GADD45G in lentiviral vectors significantly elongated the neurites (Figure S4).

Next, we investigated the target of this signal for microtubule extension. Here, we focused on CRMP2, which is known to promote microtubule assembly by acting on tubulin in its dephosphorylated but not its phosphorylated state (Dajani et al., 2001; Fukata et al., 2002; Ménager et al., 2004; Yoshimura et al., 2005).

We quantified the level of phospho-CRMP2 in hiPSC-NS/PC-derived neurons by western blotting and found that the amount of phospho-CRMP2 was decreased by the addition of RK-682 (Figure 4G). This analysis revealed that neurite outgrowth in the RK-682-treated group was caused by the dephosphorylation of CRMP2, which promoted microtubule polymerization. Taken together, our findings indicate that a kinase cascade starting from GADD45G, which adds phosphate groups, promotes CRMP2-induced microtubule polymerization and neurite outgrowth (Figure 4H).

We then examined whether blocking GADD45G/p38 MAPK/CDC25B signaling at downstream points could modulate the phenotype of neurite outgrowth. To measure neurites, neurospheres were cultured with SB203580 and GSIs for 14 days to induce neuronal differentiation and then immunostained for β-III tubulin or tau. Neurite outgrowth was suppressed by inhibition of the p38 signal (Figures 4I, 4J, S5A, and S5B), indicating that phospho-p38 induces the phosphorylation of CDC25B, thereby leading to neurite extension.

Furthermore, we investigated the effect of siRNA knockdown of the downstream effector CDC25B (Figure S5E). Fourteen days of culture with siRNA and GSIs resulted in the differentiation of neurospheres into neurons, and the knockdown of CDC25B suppressed neurite outgrowth despite GSI treatment (Figures 4K, 4L, S5C, and S5D). On the other hand, knockdown of *CDC25B* did not inhibit differentiation into neurons (Figures 4K and 4L high-magnification image). These results suggest that *CDC25B* induces neurite extension without affecting neuronal differentiation. We also found that the degree of phosphorylation of CRMP2 in the neurospheres derived from iPSCs without any pretreatment was higher in the group with siRNA against CDC25B than in the control group without siRNA (Figure S5F), suggesting that phosphorylation status of CRMP2 is crucial in CDC25B-induced neurite extension as shown in Figure 4H.

### The GADD45G/p38 MAPK/CDC25B signaling pathway does not contribute to microtubule stability

We also evaluated whether this signaling pathway contributes to the stability of microtubules by quantifying α-tubulin acetylation (Lys40), which is an index of microtubule stabilization (Roll-Mecak, 2020).

DMSO or RK-682 was administered 5 days after the induction of neuronal differentiation from hiPSC-NS/PCs, and the degree of acetylation triggered by RK-682 was analyzed by immunoblotting. There was no difference in the degree of acetylation of α-tubulin (Lys40) between the control group and the RK-682-treated group (Figure S6A). In addition, the degree of acetylation of α-tubulin in similarly treated neurons was quantified by immunostaining. The degree of acetylation did not change between the control group and the RK-682-treated group (Figure S6B).

Taken together, the data indicate that the neurite outgrowth effect of RK-682 treatment is more likely to be mediated by promotion of the microtubule polymerization reaction rather than microtubule stability.

### Enhanced p38 MAPK/CDC25B signaling is effective for neurite outgrowth even after neuronal differentiation from neural stem/progenitor cells (NS/PCs)

**Figure 5:**
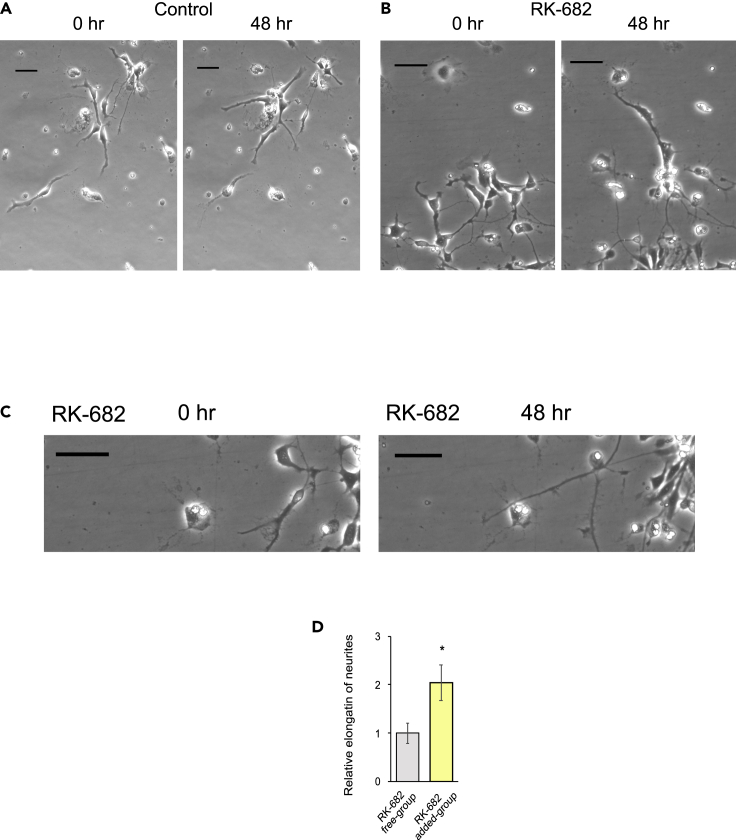
Increased phospho-p38/phospho-CDC25B expression after neuronal differentiation further increases neurite length (A and B) On day 5 after the induction of neuronal differentiation from hiPSC-NS/PCs, DMSO (A) or RK-682 (B) was added, and neurons were cultured for 48 h. Representative images obtained by the time-lapse method are shown. Scale bars: 50 μm. (C) Magnified images of neurites elongated by RK-682. Scale bars: 50 μm. (D) RK-682 was added to the neurons, and the degree of neurite outgrowth was measured and quantified 2 days later. All neurons in four visual fields were quantified for each experiment (n = 3; p = 0.0494).Statistical analyses were performed with unpaired two-tailed Student’s t-tests. The values in the bar graphs represent the means ± SEs. ∗p < 0.05.

In the above experiments, we studied the effect of RK-682 on heterogeneous cell populations during the differentiation of iPSC-derived NS/PCs into neurons by transferring neurospheres into an adherent culture system; therefore, we were not able to determine the specific effect on postdifferentiated neurons. To investigate the effects of RK-682-mediated enhancement of p38 MAPK/CDC25B signaling on postdifferentiated neurons, we examined neurite growth following the differentiation of single hiPSC-NS/PCs into neurons. RK-682 was administered 5 days after the induction of neuronal differentiation, and the degree of neurite extension triggered by RK-682 was observed by time-lapse imaging for 48 h. After the addition of RK-682, neurite extension was significantly increased (Figures 5A–5D and Videos S1 and S2).

Video S1. Time-lapse movie, related to Figure 5 On day 5 after the induction of neuronal differentiation, DMSO was added, and the neurons were cultured for 48 h.

Video S2. Time-lapse movie, related to Figure 5 On day 5 after the induction of neuronal differentiation, RK-682 was added, and the neurons were cultured for 48 h.

### GADD45G is highly expressed in developing human cerebral samples, consistent with the neuron production period

We next assessed *GADD45G* expression in the human cerebrum *in vivo*. Bulk RNA-seq data of human cerebral samples were obtained from the BrainSpan Atlas of the Developing Human Brain (http://www.brainspan.org) to analyze the expression of *GADD45G* in human brains from the fetal to the adult period.

**Figure 6:**
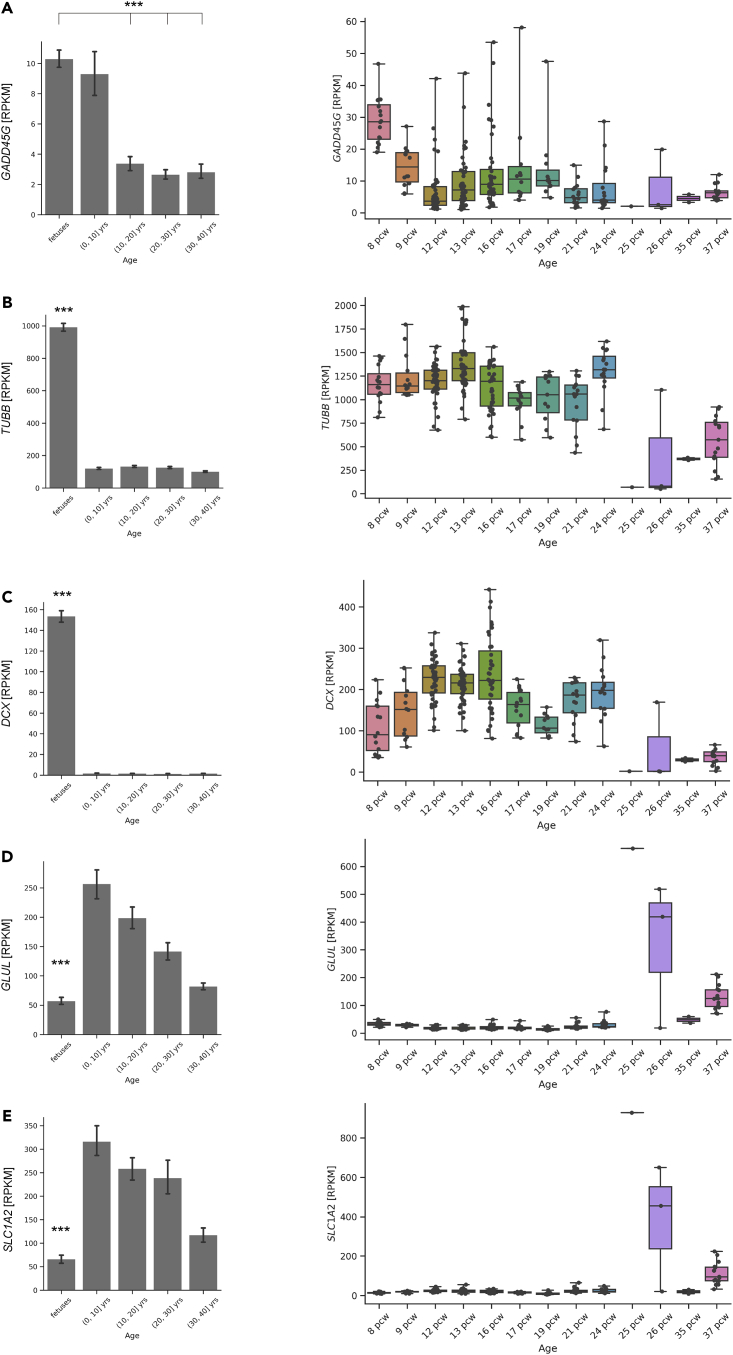
*GADD45G* is highly expressed during neurodevelopment, especially in the early fetal human brain (A–E) In the bar charts, the *GADD45G* (A)*, TUBB* (B)*, DCX* (C)*, GLUL* (D), and *SLC1A2* (E) expression levels (RPKM values) were compared among the following generations: fetuses and patients aged 0 to 10 years, 10 to 20 years, 20 to 30 years, and 30 to 40 years. In the box charts, fetuses were stratified by postconceptional weeks (pcw), and each dot represents each sample’s detailed expression (RPKM value) of the gene.Bulk RNA-seq data from human brains were obtained from the BrainSpan Atlas of the Developing Human Brain (http://www.brainspan.org). Statistical analyses were performed with one-way ANOVA followed by Steel-Dwass post hoc tests. The values in the bar graphs represent the means ± SEs. ∗∗∗p < 0.001.

The expression of *GADD45G* was significantly higher in the fetal brain and in the brains of humans up to 10 years of age than in the brains of older humans (Figure 6A). Moreover, the expression of *GADD45G* also differed depending on the fetal stage, as it tended to be higher in the early embryonic period than in the late embryonic period (Figure 6A).

During this period of high *GADD45G* expression, the neuronal markers TUBB and DCX were expressed at high levels, whereas the astrocytic markers SLC1A2 (Desilva et al., 2012) and GLUL (Li et al., 2019) were expressed at significantly reduced levels (Figures 6B–6E). The high expression of GADD45G in the early embryonic period, when neurogenesis is active, suggests that GADD45G may be involved in the functional and morphological aspects of neurons, including neurite outgrowth.

## Discussion

**Figure 7:**
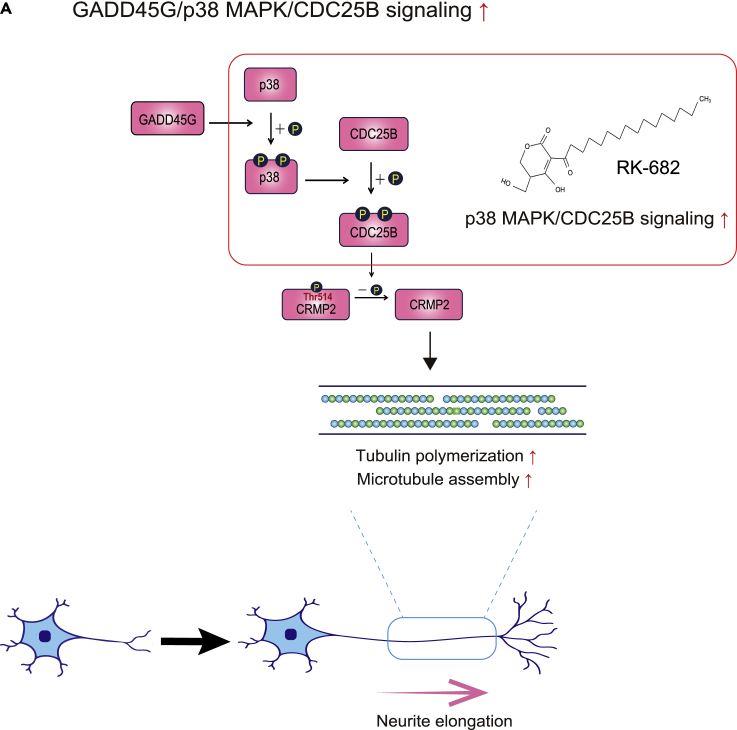
Conceptual diagram of the research results (A) Schematic of the major results. The GADD45G/p38 MAPK/CDC25B signaling pathway promotes microtubule polymerization via dephosphorylation of CRMP2 and elongates neurites in human neurons. RK-682 promotes microtubule polymerization and elongates neurites by increasing the activity of p38 MAPK/CDC25B signaling.

The present study revealed a novel role of the GADD45G/p38 MAPK/CDC25B signaling pathway, which facilitates neurite outgrowth via promotion of microtubule polymerization (Figure 7A). CDC25B is generally known to regulate centrosomal microtubule nucleation (Boutros et al., 2007). However, microtubule nucleation in neurites occurs in a noncentrosomal mode (Stiess et al., 2010). The present study found that CDC25B is also involved in microtubule polymerization in a noncentrosomal form during neurite outgrowth.

This study also revealed that the action of CDC25B in neurite outgrowth is regulated by its phosphorylation by p38 MAPK. Phosphorylation of CDC25A and CDC25B has been reported to promote G2/M transition in mammalian cells (Wu et al., 2014), but CDC25 family members have been reported to be degraded by phosphorylation in another study (Karlsson-Rosenthal and Millar, 2006). Although some reports suggest that the degradation of CDC25A by nongenomic damage-related stress is caused by p38, the degradation of CDC25B is caused mainly by c-Jun N-terminal kinase and proteasomes (Isoda et al., 2009; Uchida et al., 2009). Thus, these results suggest that the degradation and activation of these molecules are caused by different mechanisms (mediated by different MAPKs), even among CDC25 family members.

Moreover, CDC25B is required for neural differentiation during mouse development (Peco et al., 2012), and a study on chick neural progenitor cells revealed that CDC25B contributes to neural differentiation independently of its effect on the cell cycle (Bonnet et al., 2018). Another study demonstrated that the promotion of neurite growth by PRG3 requires CDC25 during mouse development (Broggini et al., 2016). These reports support our present findings.

In Alzheimer’s disease, CDC25B activity increases the abnormal expression of Cdc2/cyclin B, resulting in various downstream indicators of mitotic events and eventually leading to neurodegeneration (Vincent et al., 2001). However, CDC25B lacks the responsiveness of Cdc2/cyclin B in healthy neurons (Vincent et al., 2001). The present results and those in several other reports suggest that CDC25B does not necessarily have adverse effects on neuronal cells under physiological conditions.

The present study showed that *GADD45G* expression was increased in developing human fetal brain samples, showing gene expression patterns matching those in hiPSC-NS/PCs treated with GSIs. Consistent with this, a database identified *GADD45G* as being upregulated upon the neuronal differentiation and maturation of immature neurons derived from embryonic stem (ES) cells (van de Leemput et al., 2014), indicating that this pathway may become activated as neurons develop in the early fetal stage.

Taken together with other research results, our findings indicate that the crisp expression regulation of *GADD45G* may contribute to the specificity of the human brain. It has also been reported that the enhancer sequence of *GADD45G*, which is conserved in nonhuman mammals, including chimpanzees, is specifically deleted in the human genome (hCONDEL) (McLean et al., 2011). This deleted sequence is thought to promote the expression of *GADD45G* in neural progenitor cells in the fetal brains of nonhuman mammals. It is speculated that this deletion reduced the expression of *GADD45G* in human NS/PCs, resulting in a more proliferative trait in the human brain, which led to the enlargement of the human brain during evolution. Thus, an attractive hypothesis is that the upregulation of *GADD45G* in the neuronal lineage that takes place both *in vitro* and *in vivo* plays an important role in the switch from the proliferative state of progenitor cells to the neurite outgrowth state in human postmitotic neurons. The results of our experiments also support this hypothesis. GSIs promote the differentiation of proliferative neural progenitor cells into postmitotic neurons (Okubo et al., 2018), and the elevated expression of *GADD45G* in GSI-treated neurospheres may play an important role in the neurite outgrowth. However, the precise mechanism of human-specific regulation of *GADD45G* in neuronal development remains to be elucidated.

CRMP2 is the most highly expressed protein among the CRMP family in the brain (Charrier et al., 2003; Wang and Strittmatter, 1996), and it is the one that has been analyzed the most. The function of CRMP2 protein differs depending on the site of phosphorylation or dephosphorylation.

In particular, phosphorylation of CRMP2 Thr514 is known to be involved in neurite outgrowth, and dephosphorylation of Thr514 tightens the bond between tubulin and CRMP2, leading to microtubule polymerization (Yoshimura et al., 2005). The anti-phosphorylation antibody used in our experiments is specific for Thr514.

It has been reported that RK-682 does not affect the phosphorylation of CDC25B (Hamaguchi et al., 1995), suggesting that CDC25B is located downstream of p38 signaling. Knockdown of CDC25B siRNA resulted in higher levels of CRMP2 phosphorylation compared to the control (non-knockdown) group, suggesting that CDC25B may alter the phosphorylation state of CRMP2 either directly or through signals located further downstream.

### Limitations of the study

One thing that must be considered is that treatment of hiPSC-derived neurospheres with GSI upregulates the expression of other genes than we originally focused on, such as DCX (Figure 1B). DCX, which is often used as a neuronal marker, stabilizes microtubules by cross-linking to microtubule protofilaments (Moorse et al., 2006). In the signaling pathway discovered in this study, CRMP2 promotes microtubule polymerization, though it is possible that DCX and other latent genes act in association with this signaling pathway to elongate microtubules. It is necessary to investigate the alternative cascades in the future.

This discovery in the present study was derived from iPSC-NS/PCs and premature neurons in the developmental phase. On the other hand, neurites regress, and brain dysfunction increases during aging and in the contexts of neurodegenerative diseases (Petratos et al., 2008; Scheibel et al., 1975). Whether the GADD45G/p38 MAPK/CDC25B signaling pathway is involved in neuronal regression because of aging should be investigated in the future. Moreover, in terms of regenerative medicine, clarifying the mechanism underlying neurite outgrowth might aid in the development of treatments for neurodegenerative diseases.

## STAR★Methods

### Key resources table

**Table:** 

REAGENTS and RESOURCES	SOURCE	IDENTIFIER
**Antibodies**		

Mouse β-III tubulin antibody	Sigma-Aldrich	Cat# T8660; RRID:AB_477590
Rabbit α-tubulin antibody	Cell Signaling Technology	Cat# 2144; RRID:AB_2210548
Rabbit anti-tau antibody	Dako	Cat# A0024; RRID:AB_1001372
Rabbit p38 MAPK antibody	Cell Signaling Technology	Cat# 8690; RRID:AB_10999090
Rabbit phospho-p38 MAPK antibody	Cell Signaling Technology	Cat# 4511; RRID:AB_2139682
Rabbit phospho-CDC25B antibody	Thermo Fisher Scientific	Cat# PA5-104568; RRID:AB_2816043
Goat anti-CDC25B antibody	R&D systems	Cat# AF1649; RRID:AB_2075440
Rabbit GADD45G polyclonal antibody	Thermo Fisher Scientific	Cat# PA5-21921; RRID:AB_11152673
Rabbit phospho-CRMP2 (Thr514) antibody	Affinity Bioscience	Cat# AF3459; RRID:AB_2834897
Mouse monoclonal anti-β-Actin antibody	Sigma-Aldrich	Cat# A1978, RRID:AB_476692
Acetyl-α-tubulin (Lys40) (D20G3) XP Rabbit monoclonal antibody	Cell Signaling Technology	Cat# 5335; RRID:AB_10544694
Goat anti-rabbit IgG (H + L) Alexa Fluor 488	Thermo Fisher Scientific	Cat# A-11034; RRID:AB_2576217
Goat anti-mouse IgG (H + L) Alexa Fluor 555	Thermo Fisher Scientific	Cat# A-21424; RRID:AB_141780
Goat anti-mouse IgG antibody, (H + L) HRP conjugate	Jackson ImmunoResearch	Cat# 115-035-166; RRID:AB_2338511
Goat anti-rabbit IgG antibody, (H + L) HRP conjugate	Jackson ImmunoResearch	Cat# 111-035-144; RRID:AB_2307391
Goat anti-goat IgG antibody, (H + L) HRP conjugate	Jackson ImmunoResearch	Cat# 705-035-147; RRID:AB_2313587

**Chemicals, peptides, and recombinant proteins**		

DAPT	Sigma-Aldrich	D5942-5MG
Compound 34	Santa Cruz	sc-391108A
RK-682, protein tyrosine phosphatase (PTP) inhibitor	Abcam	ab141730
HiPerFect Transfection Reagent	QIAGEN	301705
Recombinant Human FGF-basic	PeproTech	100-18B
Animal-free Recombinant Human EGF	PeproTech	AF-100-15
B-27™ Supplement (50X), serum-free	Thermo Fisher Scientific	17504-044
Penicillin-Streptomycin Mixed Solution	Nacalai Tesque	26253-84
TrypLE™ Select (1X), no Phenol Red	Thermo Fisher Scientific	12563029
D-MEM/Ham's F-12	Wako	042-30795
SB203580	Cell Signaling Technology	5633S
Lysis Buffer	Sigma-Aldrich	C0616
Halt™ Phosphatase Inhibitor Cocktail	Thermo Fisher Scientific	78420
Hoechst 33258	Sigma-Aldrich	B2883
ECL Prime Western Blot Detection Reagent	GE Healthcare	RPN2236
SB431542	Tocris	301836-41-9
LDN193189	StemRD	1062368-24-4
B27 Supplement (50X), minus vitamin A	Thermo Fisher	12587001
Y-27632 (Calbiochem)	Nacalai Tesque	08945-71
Retinoic acid	Sigma-Aldrich	R2625-1G
CHIR 99021	Stemgent	REPROCELL
SB203580	Cell Signaling Technology	5633S

**Experimental models: Cell lines**		

Human iPSC (hiPSC) line 201B7	CiRA (Kyoto University)	N/A
Human iPSC (hiPSC) line 414C2	CiRA (Kyoto University)	N/A

**Software and algorithms**		

Matplotlib 3.3.3	Hunter (2007)	https://github.com/matplotlib/matplotlib
Numpy 1.19.5	Harris et al. (2020)	https://github.com/numpy/numpy
Pandas 1.2.0	McKinney (2010)	https://github.com/pandas-dev/pandas
Seaborn 0.11.1	Waskom (2021)	https://github.com/mwaskom/seaborn
Scikit-learn 0.24.0	Pedregosa et al. (2011)	https://github.com/scikit-learn/scikit-learn
Scipy 1.6.2	Jones et al	https://github.com/scipy/scipy

**Other**		

Human embryonic stem cell (hESC) medium	Reprocell	RCHEMD001
Corning® 100 mm Ultra-Low Attachment Culture Dish	Corning	3262
Chamber slide glasses	IWAKI	5732-008
Silencer® Select SiRNA (negative control)	Thermo Fisher Scientific	4390844
Silencer® Select SiRNA (CDC25B)	Thermo Fisher Scientific	4390824
Silencer® Select SiRNA (GADD45G)	Thermo Fisher Scientific	4392420

### Resource availability

#### Lead contact

Further information and requests for resources and reagents should be directed to and will be fulfilled by the Lead Contact, Hideyuki Okano (hidokano@keio.jp).

#### Materials availability

This study did not generate new unique reagents.

### Experimental model and subject details

#### Culture of undifferentiated hiPSCs

The hiPSC lines 201B7 (CiRA; Kyoto University) and 414C2 (CiRA; Kyoto University) were cultured with mitomycin C-treated SNL murine fibroblast feeder cells in standard human embryonic stem cell (hESC) medium (Reprocell, RCHEMD001) containing 0.5% penicillin-streptomycin (Nacalai Tesque, 26253-84) and 4 ng/mL fibroblast growth factor 2 (FGF-2) (PeproTech, 100-18B) in an atmosphere containing 5% CO_2_.

#### Formation of neurospheres

hiPSCs (414C2 line) were cultured for 12 days in adhesion cultures with mouse embryonic fibroblasts and then allowed to form embryonic bodies in floating culture for 30 days. Aggregated cells were differentiated into NS/PCs derived from hiPSC-NS/PCs using various factors during each day of the incubation period (Okada et al., 2008).

hiPSCs (201B7 line) were pretreated for 6 days with 3 μM SB431542 (Tocris, 301836-41-9) and 150 nM LDN193189 (StemRD, 1062368-24-4). The cells were then dissociated and seeded at a density of 1×10^5^ cells per milliliter in ultra-low-attachment culture dishes (Corning) in neuronal induction medium consisting of medium hormone mix (MHM) (Okada et al., 2008) supplemented with 2% B27 supplement without vitamin A (Thermo Fisher, 17504-044), 20 ng/mL FGF-2, 10 μM Y27632 (Nacalai Tesque, 08945-71), 1 μM retinoic acid (RA; Sigma, R2625-1G), 3 μM CHIR 99021 (Reprocell, 04-0004) and 10 μM SB431542 (Calbiochem, 301836-41-9) in a hypoxic and humidified atmosphere (4% O_2_, 5% CO_2_) for 6 days. The formed neurospheres were passaged by dissociation into single cells and then cultured in slightly modified neuronal induction medium, MHM supplemented with 2% B27 without vitamin A, 20 ng/mL FGF-2, 10 μM Y27632, and 1 μM RA for 6 days under 4% O_2_ (hypoxic) conditions.

#### Differentiation of neurons derived from hiPSC-NS/PCs (neurospheres)

hiPSC-NS/PCs were plated onto poly-L-ornithine/laminin-coated chamber slide glasses (Iwaki). The cells were incubated in MHM medium supplemented with 2% B27 and 1% penicillin/streptomycin in a humidified atmosphere at 37°C for 14 days.

### Method details

#### Lentivirus production

Control lentiviral vectors expressing Venus (Kase et al., 2019) were produced by transient transfection of Lenti-X 293T cells (Takara Bio, Inc., 632180) with the lentivirus constructs, pCMV-VSV-G-RSV-Rev and pCAG-HIVgp48 using GeneJuice® Transfection Reagent (Merck Millipore, 70967-6CN) according to the manufacturer’s instructions. High-titer (>109 IFU/mL) concentrated stocks prepared by ultracentrifugation and resuspension in PBS were used to obtain efficient infections. GADD45G overexpressing lentiviral vectors; pLV[Exp]-EGFP-EF1A>hGADD45G were synthesized by VectorBuilder.

#### Lentiviral infection

Neurospheres were transduced with lentiviral vectors (MOI = 3).

#### Immunostaining

For immunocytochemistry, samples (neurospheres or neurons: derived from the 201B7 line) were plated onto poly-L-ornithine/fibronectin-coated chamber slide glasses (Iwaki) and fixed in 4% PFA/PBS for 30 min at room temperature. The slides were rinsed with PBS three times and permeabilized with 0.3% Triton X-100/PBS for 5 min at room temperature. After blocking with Blocking One (Nacalai Tesque, 03953-95) for 15 min at room temperature, the slides were incubated at 4°C overnight with the following antibodies: rabbit anti-α-tubulin (Cell Signaling Technology, 2144; 1:500), mouse anti-β-III tubulin (Sigma-Aldrich, T8660-2ML; 1:500), rabbit anti-tau (Dako, A0024; 1:500), rabbit anti-p38 MAPK (Cell Signaling Technology, 8690; 1:500), rabbit anti-phospho-p38 MAPK (Cell Signaling Technology, 4511; 1:500), rabbit anti-phospho-CDC25B (Thermo Fisher Scientific, PA5-104568; 1:500), and rabbit anti-acetyl-α-tubulin (Lys40) (Cell Signaling Technology, 5335; 1:500). After washing three times with PBS, the samples were incubated with secondary antibodies conjugated to Alexa 488 (Thermo Fisher Scientific, A-11034) or Alexa 555 (Thermo Fisher Scientific, A-21424; 1:500) for 60 min at room temperature and then subjected to nuclear counterstaining with Hoechst 33258 (Sigma-Aldrich, B2883; 10 μg/mL). The samples were analyzed with an all-in-one fluorescence microscope (BZ-700 or BZ-800, Keyence).

#### Treatment of neurospheres with GSIs and RK-682

The GSIs N-[N-(3,5-difluorophenacetyl)-*l*-alanyl]-S-phenylglycine t-butyl ester (DAPT; Sigma-Aldrich, D5942-5MG), (2S,3R)-3-(3,4-difluorophenyl)-2-(4-fluorophenyl)-4-hydroxy-N-((3S)-2-oxo-5-phenyl-2,3-1H-benzo[e][1,4]diazepin-3-yl)butyramide (Compound 34: Santa Cruz, sc-391108A), and (2R)-4-hexadecanoyl-3-hydroxy-2-(hydroxymethyl)-2H-furan-5-one (RK-682) were dissolved in DMSO. The neurospheres were cultured for 24 h with either 10 μM DAPT, 2 μM Compound 34, or 20 μM RK-682.

#### RNA-seq of neurospheres

Neurosphere sequencing was performed by TaKaRa Bio, Inc. (Kusatsu, Japan). Purified RNA was resuspended in Clontech buffers for mRNA amplification by 5′ template switching PCR with a Clontech SMART-Seq v4 Ultra Low Input RNA Kit according to the manufacturer’s instructions. The amplified cDNA was fragmented and linked with dual-indexed barcodes using Illumina Nextera XT DNA Library Prep Kits. The libraries were validated using an Agilent 4200 TapeStation, pooled and sequenced on the Illumina NovaSeq 6000 platform.

#### Visualization of the RNA-seq results

Transcripts per million (TPM) values were selected as representative data for gene expression and converted into log_2_(TPM+1) values for normalization using Numpy and Pandas. Heat maps were created by using Matplotlib and Seaborn. The RNA-seq data were processed by PCA with Scikit-learn to reduce dimensionality and calculate the components in the eigenvectors of the principal components. The number of dimensions was determined from the values of cumulative contribution, and the threshold was set to 80%. For clustering analysis, k-means clustering was performed by using Scikit-learn, and elbow plots were made by using Matplotlib, Numpy, Pandas, and Scikit-learn. Scatterplots and a bar chart were created with Matplotlib and Pandas. For all procedures that required pseudorandom numbers, the random seeds were fixed to 0. The RNA-seq data (expression levels provided as RPKM values) shown in Figure 4 were downloaded from BrainSpan and processed with Pandas, Numpy, Matplotlib, and Seaborn and are summarized in bar charts or box charts. All the libraries mentioned above are available in the Python programming language.

#### Treatment with siRNA

Silencer® Select siRNA (negative control, Thermo Fisher Scientific, catalog# 4390844), Silencer® Select siRNA (GADD45G) (Thermo Fisher Scientific, catalog# 4392420, assay ID s21450), and Silencer® Select siRNA (CDC25B) (Thermo Fisher Scientific, catalog# 4390824, assay ID s2754) were mixed with HiPerFect Transfection Reagent (Qiagen 301705) and cultured with the neurospheres and neurons at a final concentration of 5 nM.

#### Western blot analysis

A goat anti-CDC25B antibody (R&D Systems, AF1649; 1:500), a rabbit anti-acetyl-α-tubulin (Lys40) antibody (Cell Signaling Technology, 5335; 1:500), a rabbit phospho-CRMP2 (Thr514) antibody (Affinity Bioscience, AF3459; 1:500) and a mouse anti-β actin antibody (Sigma-Aldrich, A1978; 1:500), rabbit anti-p38 MAPK (Cell Signaling Technology, 8690; 1:500), rabbit anti-phospho-p38 MAPK (Cell Signaling Technology, 4511; 1:500), were used as primary antibodies, and anti-mouse IgG (Jackson ImmunoResearch, 115-035-166) (1:1000), anti-goat IgG (Jackson ImmunoResearch, 705-035-147) (1:1000) and anti-rabbit IgG (Jackson ImmunoResearch, 111-035-144) (1:1000) were used as secondary antibodies. Neurospheres from each condition were lysed in lysis buffer (Sigma-Aldrich, C0616) with Halt™ Phosphatase Inhibitor Cocktail (Thermo Fisher Scientific, 78420). Loading samples were prepared and separated by SDS-PAGE (4–15%) and processed for western blot analysis with a standard protocol. The signals were detected using ECL Prime Western Blot Detection Reagent (GE Healthcare, RPN2236) and visualized using LAS-4000 mini (Fujifilm). For quantification, the inbuilt Gels function was used to first convert the band intensities into histograms, from which the area under the curve was measured using the Wand tool, and the relative expression between control and treated samples was calculated using ImageJ software (NIH).

### Quantification and statistical analysis

All the statistical details of the experiment can be found in the legend of each figure. For each statistical analysis, at least three independent experiments were carried out.

Statistical significance was determined with two-tailed Student’s t-tests or one-way ANOVA followed by Tukey-Kramer or Steel-Dwass post hoc tests. Throughout the study, p values < 0.05 were considered to indicate statistical significance. The following significance thresholds were used throughout the study: ∗ p < 0.05, ∗∗ p < 0.01, and ∗∗∗ p < 0.001. NS: not significant. The values in the bar and line graphs represent the means ± SEs in all figures.

## Data Availability

• All data reported in this paper will be shared by the Lead contact upon request. We included an Excel file with all transcript expression levels converted to read counts as Table S1 in this paper. • This paper does not report original code. • Any additional information required to reanalyze the data reported in this paper is available from the Lead contact upon request.

## Supplemental information

Document S1. Figures S1–S6

Table S1. TPM values of all expressed genes in hiPSC-NS/PCs derived from 414C2 and 201B7 cells treated with DMSO, DAPT, or Compound 34, related to Figure 1 The TPM values of all genes detected by RNA-seq are shown in the table. The column labels express [cell type] [condition].
