# Supplementary material for: The GADD45G/p38 MAPK/CDC25B signaling pathway enhances neurite outgrowth by promoting microtubule polymerization
Source: iScience. 2022 Apr 4;25(4):104089. doi: 10.1016/j.isci.2022.104089 (PMC9042895; doi:10.1016/j.isci.2022.104089)
Supplement: Document S1. Figures S1–S6 [file mmc1.pdf]

iScience, Volume *25*

Supplemental information

The GADD45G/p38 MAPK/CDC25B signaling pathway enhances neurite outgrowth by promoting microtubule polymerization

Yoshitaka Kase, Tsukika Sato, Yuji Okano, and Hideyuki Okano

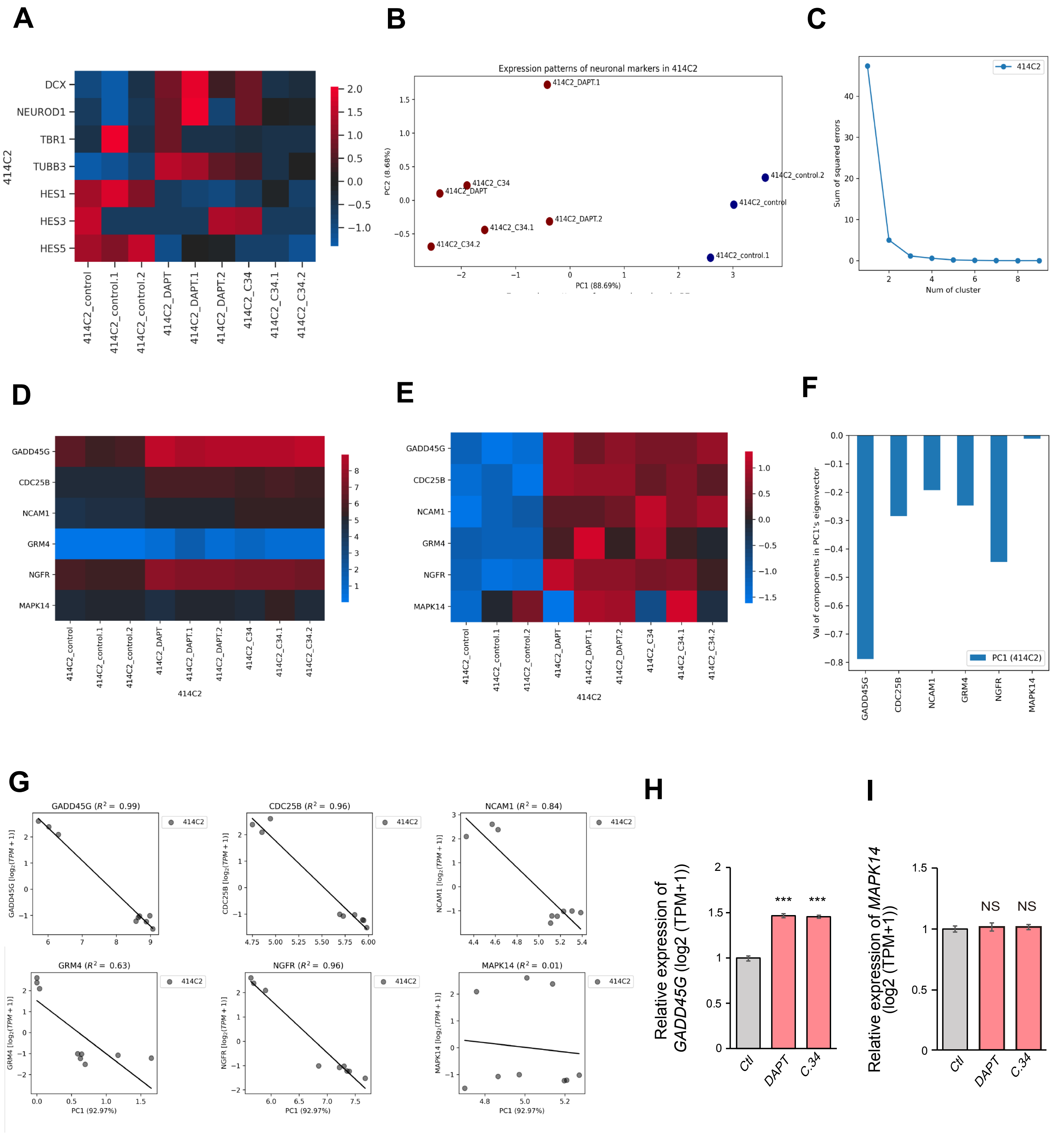
A
B
C
D
E
F
G
H
I
414C2
DCX
NEUROD1
TBR1
TUBB3
HES1
HES3
HES5
414C2_control
414C2_control.1
414C2_control.2
414C2_DAPT
414C2_DAPT.1
414C2_DAPT.2
414C2_C34
414C2_C34.1
414C2_C34.2
Expression patterns of neuronal markers in 414C2
PC1 (88.69%)
PC2 (8.68%)
Sum of squared errors
Num of cluster
GADD45G
CDC25B
NCAM1
GRM4
NGFR
MAPK14
Val of components in PC1's eigenvector
PC1 (414C2)
GADD45G ($R^2 =$ 0.99)
CDC25B ($R^2 =$ 0.96)
NCAM1 ($R^2 =$ 0.84)
GRM4 ($R^2 =$ 0.63)
NGFR ($R^2 =$ 0.96)
MAPK14 ($R^2 =$ 0.01)
PC1 (92.97%)
Relative expression of GADD45G (log2 (TPM+1))
Relative expression of MAPK14 (log2 (TPM+1))
Ctl
DAPT
C.34
***
NS

Figure S1

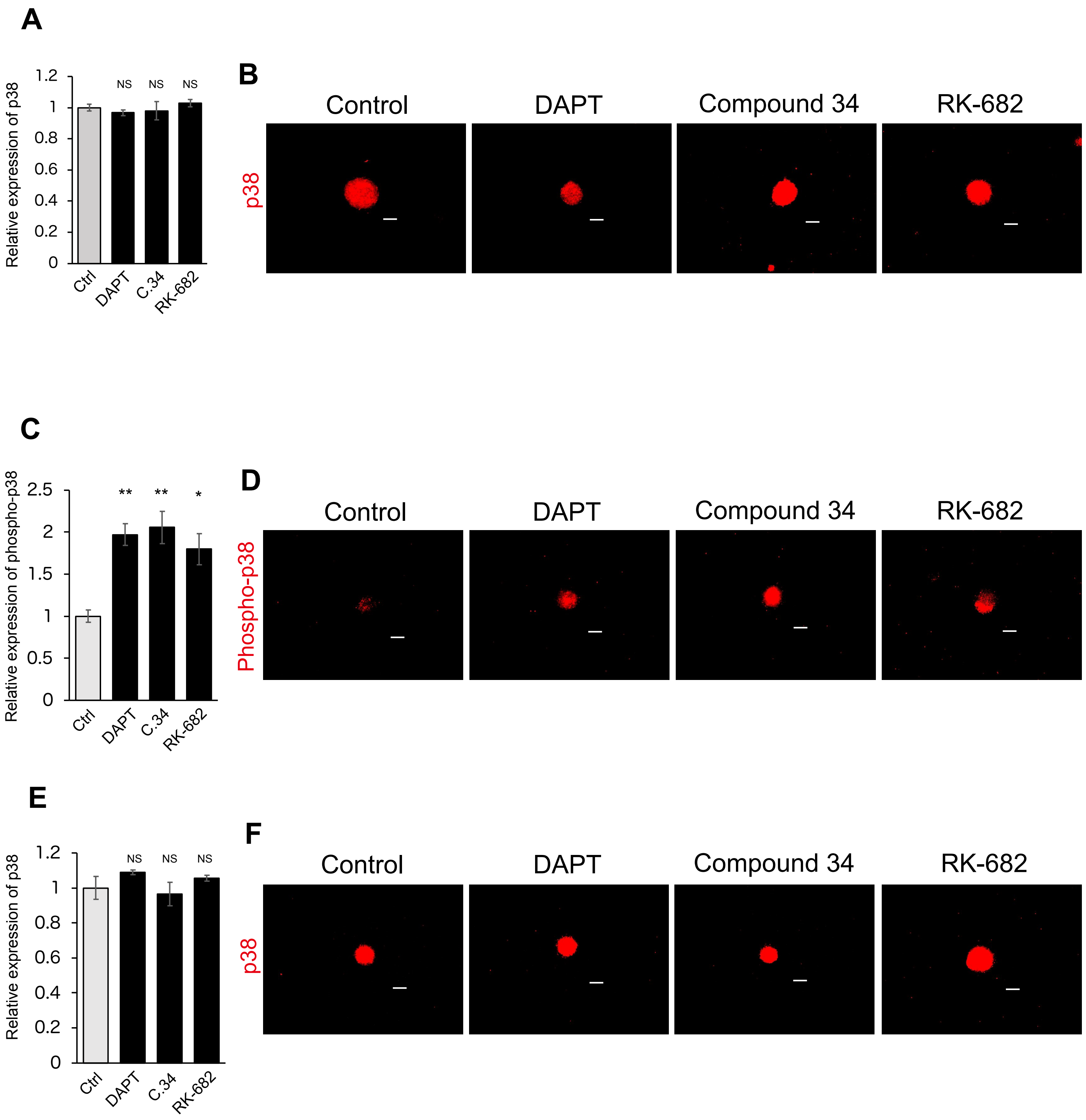
A
Relative expression of p38
1.2
1
0.8
0.6
0.4
0.2
0
NS
NS
NS
Ctrl
DAPT
C.34
RK-682
B
Control
DAPT
Compound 34
RK-682
p38
C
Relative expression of phospho-p38
2.5
2
1.5
1
0.5
0
**
**
*
Ctrl
DAPT
C.34
RK-682
D
Control
DAPT
Compound 34
RK-682
Phospho-p38
E
Relative expression of p38
1.2
1
0.8
0.6
0.4
0.2
0
NS
NS
NS
Ctrl
DAPT
C.34
RK-682
F
Control
DAPT
Compound 34
RK-682
p38

Figure S2

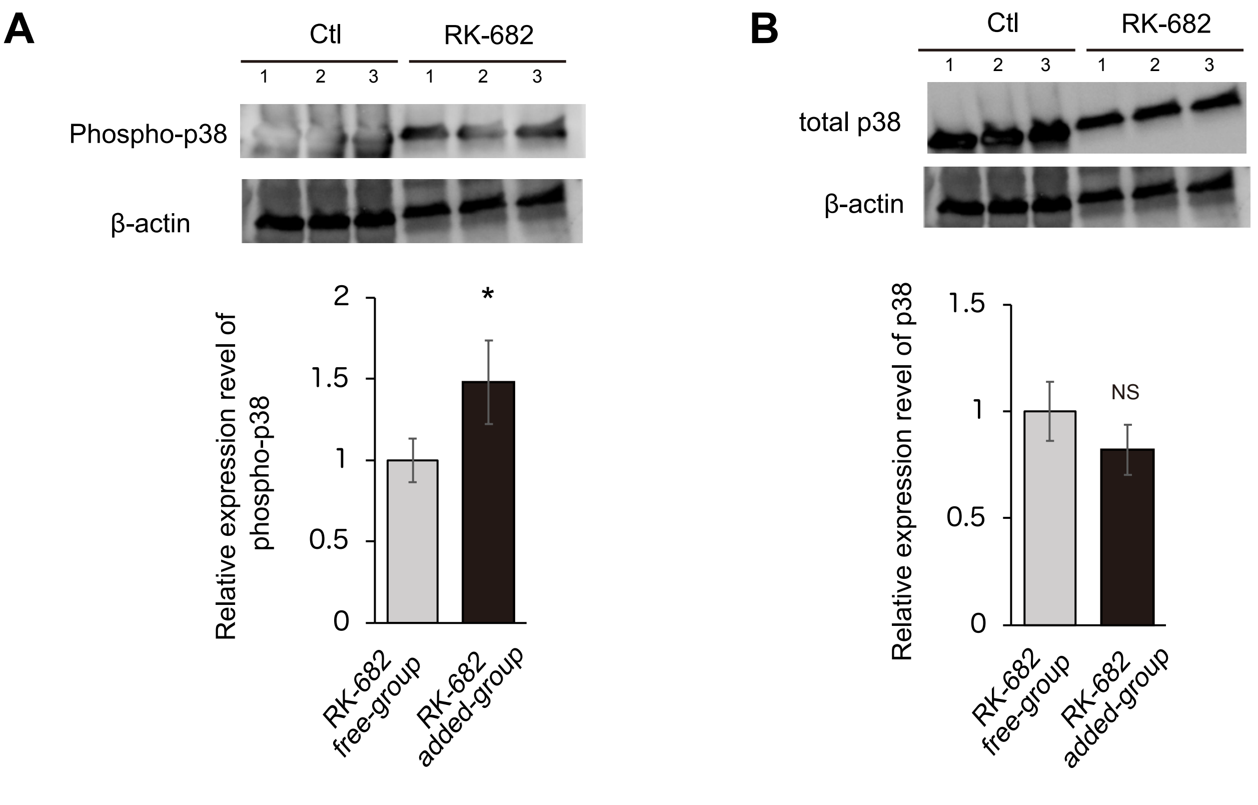
A
Ctl
RK-682
1
2
3
1
2
3
Phospho-p38
β-actin
Relative expression revel of phospho-p38
2
1.5
1
0.5
0
*
RK-682 free-group
RK-682 added-group
B
Ctl
RK-682
1
2
3
1
2
3
total p38
β-actin
Relative expression revel of p38
1.5
1
0.5
0
NS
RK-682 free-group
RK-682 added-group

Figure S3

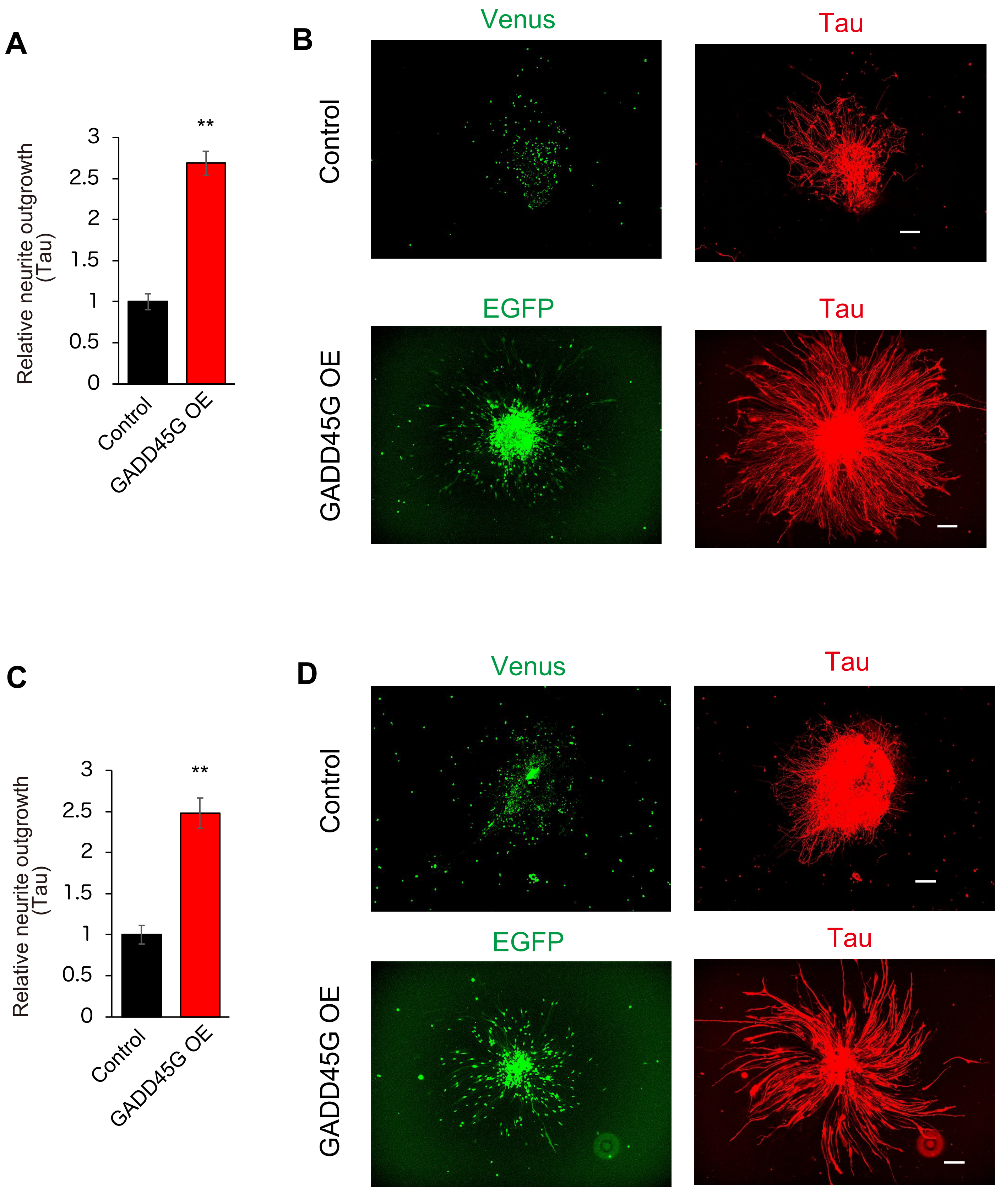
A
Relative neurite outgrowth (Tau)
3
2.5
2
1.5
1
0.5
0
**
Control
GADD45G OE
B
Venus
Tau
Control
EGFP
Tau
GADD45G OE
C
Relative neurite outgrowth (Tau)
3
2.5
2
1.5
1
0.5
0
**
Control
GADD45G OE
D
Venus
Tau
Control
EGFP
Tau
GADD45G OE

Figure S4

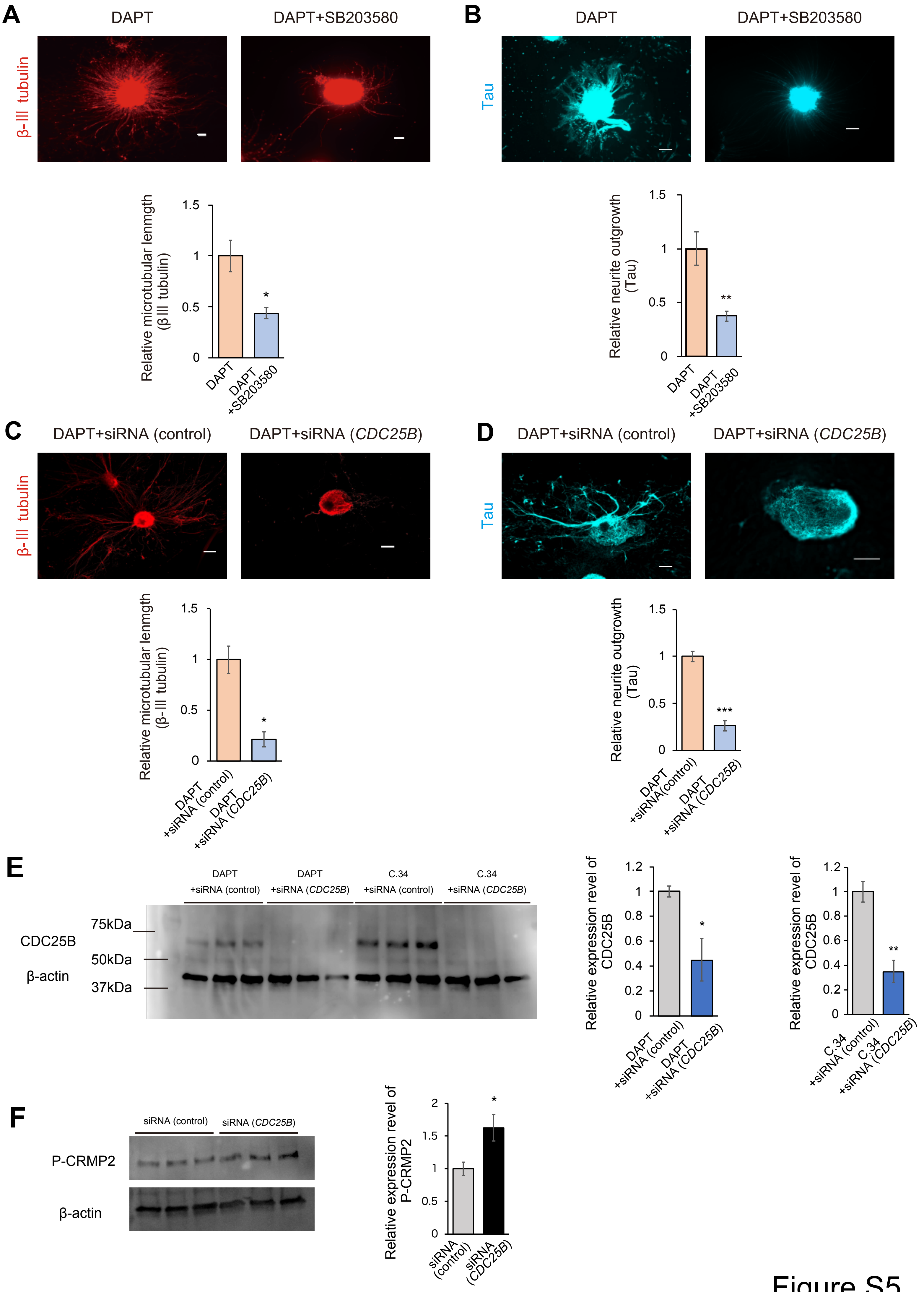
A
DAPT
DAPT+SB203580
β-III tubulin
B
DAPT
DAPT+SB203580
Tau
Relative microtubular lenmgth (β III tubulin)
Relative neurite outgrowth (Tau)
DAPT
DAPT +SB203580
C
DAPT+siRNA (control)
DAPT+siRNA (CDC25B)
β-III tubulin
D
DAPT+siRNA (control)
DAPT+siRNA (CDC25B)
Tau
Relative microtubular lenmgth (β-III tubulin)
DAPT +siRNA (control)
DAPT +siRNA (CDC25B)
Relative neurite outgrowth (Tau)
DAPT +siRNA(control)
DAPT +siRNA (CDC25B)
E
DAPT +siRNA (control)
DAPT +siRNA (CDC25B)
C.34 +siRNA (control)
C.34 +siRNA (CDC25B)
CDC25B
β-actin
75kDa
50kDa
37kDa
Relative expression revel of CDC25B
C.34 +siRNA (control)
C.34 +siRNA (CDC25B)
F
siRNA (control)
siRNA (CDC25B)
P-CRMP2
β-actin
Relative expression revel of P-CRMP2

Figure S5

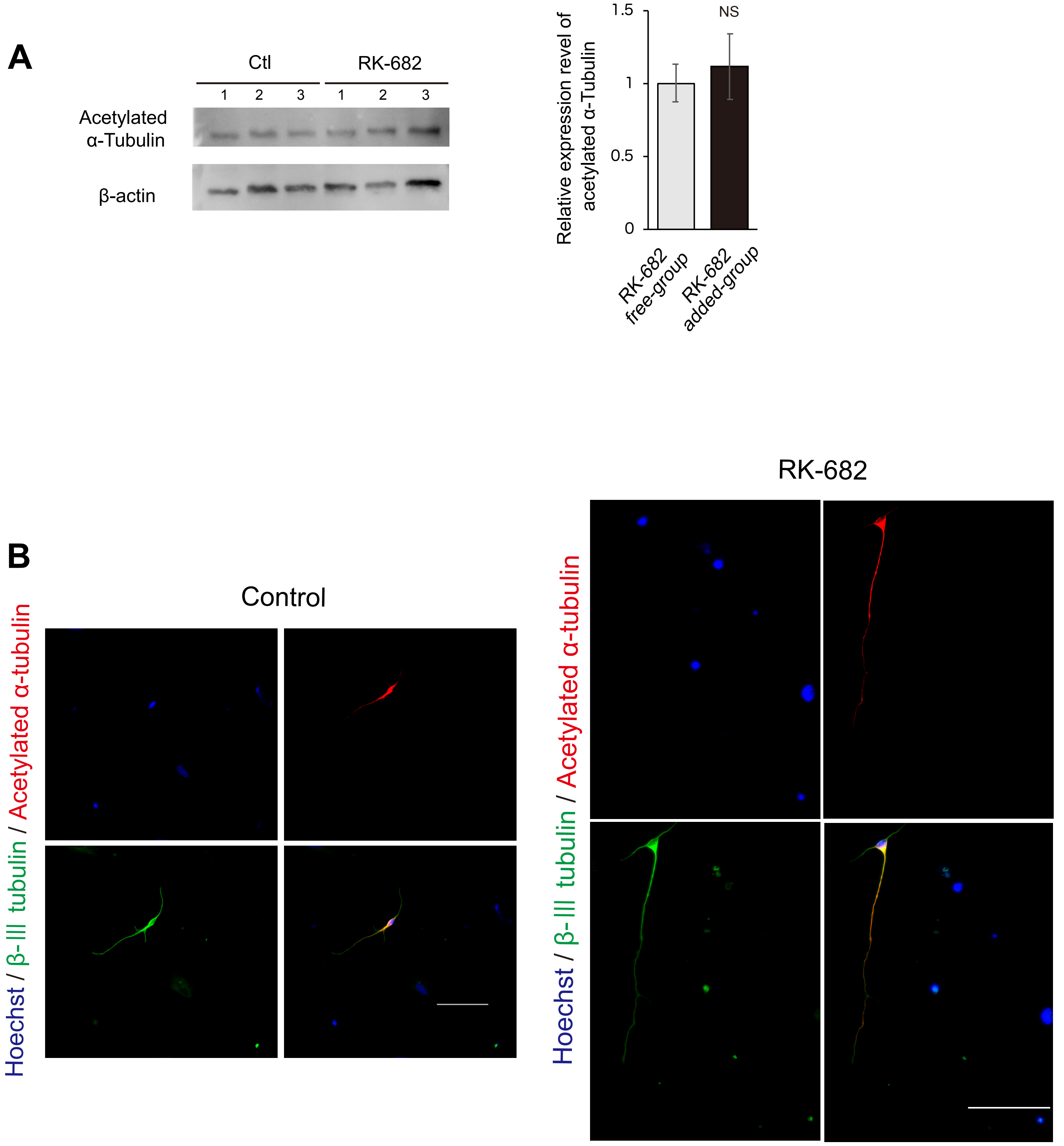
A
Ctl
RK-682
1
2
3
1
2
3
Acetylated α-Tubulin
β-actin
Relative expression revel of acetylated α-Tubulin
1.5
1
0.5
0
NS
RK-682 free-group
RK-682 added-group
B
Control
RK-682
Hoechst / β-III tubulin / Acetylated α-tubulin
Hoechst / β-III tubulin / Acetylated α-tubulin

Figure S6

Supplemental legends.

Figure S1. The potential of GSIs, which induce neuronal gene expression patterns, was confirmed even in neurospheres derived from the 414C2 cell line., Related to Figure 1.

(A) Heat map of neuronal lineage-related genes showing that DAPT and Compound 34 induced neuronal gene expression in neurospheres (derived from the 414C2 line).

(B and C) A scatter plot of the expression patterns of neuronal lineage-related genes that were decomposed by PCA is shown in Figure S1B. Each point was classified by k-means clustering, and the color of the point represents its cluster. Figure S1B suggests that the cells with DAPT or Compound 34 have similar expression patterns of neuronal lineage-related genes and are different from those of the control cells. In Figure S1C, the horizontal axis shows the number of clusters, and the vertical axis shows the sum of squared errors between the cluster centroids and each point. The “elbow” point, at which the line chart bends and plateaus, suggests that the optimal number of clusters is 2.

(D and E) In hiPSC-NS/PCs (derived from the 414C2 line) treated with DAPT or Compound 34 and control hiPSC-NS/PCs, MAPK-related genes were extracted from among all fluctuating genes, and heat maps of their gene expression levels are shown. The color of each square represents the $\log_2$(TPM+1) value in Figure S1D, while that in Figure S1E represents the Z-score value calculated from the $\log_2$(TPM+1) value of a single gene among all the samples.

(F) The $\log_2$(TPM+1) values of the genes listed in Figure S1D and S1E in the hiPSC-NS/PCs (derived from the 414C2 line) were decomposed by PCA, and the feature values were extracted as PC1. Figure S1F shows the components in the eigenvector of PC1 and suggests that *GADD45G* is the top representative gene for PC1, which explains 92.97% of the total information in the expression matrix.

(G) Linear regression models for each gene listed in Figure S1D and S1E. The objective variables (shown in the vertical axis) are the $\log_2$(TPM+1) values, the explanatory variables (shown in the horizontal axis) are PC1 calculated in Figure S1F, and the R^2 values are the coefficients of determination. The fact that the linear regression model fit PC1 well (R^2>0.26) for *GADD45G, CDC25B, NCAM1, GRM4,* and *NFGR* suggests that the expression patterns of *GADD45G* highly resembled PC1, which explained 92.97% of the total information of the MAPK-related gene expression matrix.

(H) The expression of *GADD45G* in hiPSC-NS/PCs derived from hiPSCs (414C2 line) treated with DAPT or Compound 34 was quantified (transcript level) (n=3; p<0.001, p<0.001).

Statistical analyses were performed with one-way ANOVA and Tukey-Kramer post hoc tests. The values in the bar graphs represent the means ± SEs. ***p < 0.001. C.34: Compound 34.

(I) Expression of *MAPK14* in hiPSC-NS/PCs (414C2 line) treated with DAPT or Compound 34 (transcript level) (n=3; p=0.937, p=0.935). The expression of *MAPK14* itself was not significantly changed.

Statistical analyses were performed with one-way ANOVA and Tukey-Kramer post hoc tests. The values in the bar graphs represent the means ± SEs. NS: not significant. C.34: Compound 34.

Figure S2. Treatment with DAPT, compound 34, or RK-682 increases only the level of p38 phosphorylation without changing the total p38 protein amount., Related to Figure 1 and Figure 4.

(A) Relative comparison of total p38 protein expression in hiPSC (201B7)-derived neurospheres treated with DAPT, Compound 34, or RK-682 (n=3, p=0.900, p=0.973,

p=0.936).

(B) Immunostaining image of total p38 when hiPSC (201B7)-derived neurospheres was treated with DAPT, Compound 34, or RK-682.

(C) The expression level of phosphorylated p38 in hiPSC (414C2)-derived neurospheres treated with DAPT, Compound 34, or RK-682 (n=3, p=0.00900, p=0.00500, p=0.025)

(D) Immunostaining images of phosphorylated p38 in hiPSC (414C2)-derived neurospheres treated with DAPT, Compound 34, or RK-682.

(E) The expression level of total p38 in hiPSC (414C2)-derived neurospheres treated with DAPT, Compound 34, or RK-682 (n=3, p=0.571, p=0.949, p=0.843).

(F) Immunostaining images of total p38 in hiPSC (414C2)-derived neurospheres treated with DAPT, Compound 34, or RK-682.

Statistical analyses were performed with one-way ANOVA. The values in the bar graphs represent the means ± SEs. Scale bars: 100 μm. NS: not significant. *p < 0.05, **p < 0.01. For each experiment, the experiment is performed three times and 10 neurosphere's are observed when taking the average of each group.

Figure S3. Treatment with RK-682 increases only the level of p38 phosphorylation without changing the total p38 protein amount in differentiated neuron., Related to Figure 4.

(A) Western blot analysis of phosphor-p38 between the control group and the RK-682-added group. On day 5 after the induction of neuronal differentiation, DMSO or RK-682 was added, and neurons were cultured for 48 h (n=3; p=0.0467).

(B) Western blot analysis of total p38 between the control group and the RK-682-added group. On day 5 after the induction of neuronal differentiation, DMSO or RK-682 was

added, and neurons were cultured for 48 h (n=3; p=0.166).

Statistical analyses were performed with unpaired two-tailed Student's t-tests. The values in the bar graphs represent the means ± SEs. *p < 0.05. NS: not significant. Ctl: control.

Figure S4. Overexpression of GADD45G causes neurite outgrowth., Related to Figure 2 and Figure 3.

(A) GADD45G was overexpressed in the hiPSC (201B7)-derived neurospheres and neurite outgrowth was quantified; GADD45G overexpression significantly elongated neurites (n=3; p=0.00311).

(B) Immunostaining images of tau with the addition of control vector expressing Veunus and forced expression of GADD45G (reporter: EGFP).

(C) GADD45G was overexpressed in hiPSC (414C2)-derived neurospheres and neurite outgrowth was quantified; GADD45G overexpression significantly elongated neurites (n=3; p=0.00243)

(D) Immunostaining images of tau with the addition of control vector expressing Veunus and forced expression of GADD45G (reporter: EGFP).

Statistical analyses were performed with unpaired two-tailed Student's t-tests. The values in the bar graphs represent the means ± SEs. Scale bars: 100 μm. **p < 0.01. For each experiment, the experiment is performed three times and 5 neurosphere's are observed when taking the average of each group.

Figure S5. GADD45G/p38 MAPK/CDC25B signaling promotes neurite elongation., Related to Figure 4.

(A and B) Differentiated neurons were immunostained for β-III tubulin (A) or tau (B). Neurite lengths were compared between the SB203580-treated group and the untreated group. The elongation of neurites was suppressed in the SB203580 group ($p=0.0251$, $p=0.00621$).

(C and D) After siRNA-mediated knockdown of *CDC25B*, neurites did not extend. Immunostaining for β-III tubulin (C) or tau (D) was performed to quantify neurite length. Neurite elongation was inhibited in the *CDC25B* knockdown group ($p=0.0126$, $p=0.000633$).

(E) siRNA knockdown efficiency of CDC25B as determined by western blot analysis in .

(F) Knocking out *CDC25B* with siRNA in the iPSC-derived neurospheres increased the extent of phosphorylated CRMP2, which was confirmed by western blotting.

Statistical analyses were performed with unpaired two-tailed Student's t-tests. The values in the bar graphs represent the means ± SEs. 10 neurospheres were observed for each experiment (A-D). *$p < 0.05$, **$p < 0.01$, and ***$p < 0.001$. Scale bars: 100 μm. Ctl: control.

Figure S6. p38 MAPK/CDC25B signals are not involved in microtubule stability., Related to Figure 4.

(A) Western blot analysis of acetylated α-tubulin between the control group and the RK-682-added group. On day 5 after the induction of neuronal differentiation, DMSO or RK-682 was added, and neurons were cultured for 48 h ($n=3$; $p=0.684$).

(B) Immunostaining images of β-III tubulin (green) and acetylated (Lys40) α-tubulin in

neurons (red). DMSO or RK-682 was administered 5 days after the induction of neuronal differentiation from hiPSC-NS/PCs.

Statistical analyses were performed with unpaired two-tailed Student’s t-tests. The values in the bar graphs represent the means ± SEs. Scale bars: 100 μm. *$p < 0.05$. NS: not significant.
